# Dynamics of soft nanoparticle suspensions at hard X-ray FEL sources below the radiation-damage threshold

**DOI:** 10.1107/S2052252518013696

**Published:** 2018-10-19

**Authors:** Felix Lehmkühler, Joana Valerio, Dina Sheyfer, Wojciech Roseker, Martin A. Schroer, Birgit Fischer, Kensuke Tono, Makina Yabashi, Tetsuya Ishikawa, Gerhard Grübel

**Affiliations:** a Deutsches Elektronen-Synchrotron (DESY), Notkestrasse 85, 22607 Hamburg, Germany; b The Hamburg Centre for Ultrafast Imaging, Luruper Chaussee 149, 22761 Hamburg, Germany; c Institute of Physical Chemistry, University of Hamburg, Grindelallee 117, 20146 Hamburg, Germany; d Japan Synchrotron Radiation Research Institute, 1-1-1 Kouto, Sayo-cho, Sayo-gun, Hyogo 679-5198, Japan; e RIKEN SPring-8 Center, 1-1-1 Kouto, Sayo-cho, Sayo-gun, Hyogo 679-5148, Japan

**Keywords:** XPCS, FEL, PNIPAM nanoparticles, SACLA, radiation-damage threshold, soft-matter materials

## Abstract

The application of X-ray photon correlation spectroscopy (XPCS) at free-electron laser (FEL) facilities enables, for the first time, the study of dynamics on a (sub-)nanometre scale in an unreached time range between femtoseconds and seconds. For soft-matter materials, radiation damage is a major limitation when going beyond single-shot applications. Here, an XPCS study is presented at a hard X-ray FEL on radiation-sensitive polymeric poly(*N*-isopropylacrylamide) (PNIPAM) nanoparticles. The dynamics of aqueous suspensions of densely packed silica-PNIPAM core-shell particles and a PNIPAM nanogel below the radiation-damage threshold are determined. The XPCS data indicate non-diffusive behaviour, suggesting ballistic and stress-dominated heterogeneous particle motions. These results demonstrate the feasibility of XPCS experiments on radiation-sensitive soft-matter materials at FEL sources and pave the way for future applications at MHz repetition rates as well as ultrafast modes using split-pulse devices.

## Abstract 2

The study of the dynamics of radiation-sensitive soft-matter suspensions is demonstrated by means of sequential-shot X-ray photon correlation spectroscopy below the radiation-damage threshold at the SACLA free-electron laser.

## 1. Introduction   

The study of the structure and dynamics of matter is one of the key applications of hard X-ray free-electron lasers (FEL). However, the high brilliance and ultrashort pulse lengths of state-of-the-art FELs typically leads to destruction of the sample after each pulse, a concept denoted as ‘diffract-before-destroy’ (Neutze *et al.*, 2000 ▸; Chapman *et al.*, 2006 ▸). Nowadays this is routinely used in crystallography for biological macromolecules at FEL instruments (Barty *et al.*, 2013 ▸). On the other hand, radiation damage is a severe problem in experiments beyond single-shot studies. One example is the study of dynamics on time scales between femtoseconds and seconds. This is covered by X-ray photon correlation spectroscopy (XPCS) based on coherent X-ray scattering.

XPCS has become a well established technique to study dynamics on length scales from several 100 nm down to atomic dimensions. In XPCS experiments, the dynamics are accessed from correlations of coherent diffraction patterns, otherwise known as speckle patterns. Recent studies cover ageing in metallic glasses (Giordano & Ruta, 2016 ▸; Evenson *et al.*, 2015 ▸) and nanoscale dynamics in amorphous ice (Perakis *et al.*, 2017 ▸). Comprehensive overviews of XPCS can be found in various review publications (Grübel & Zontone, 2004 ▸; Grübel *et al.*, 2008 ▸; Shpyrko, 2014 ▸; Madsen *et al.*, 2015 ▸; Sandy *et al.*, 2018 ▸). For soft-matter systems XPCS is used whenever the application of light-scattering methods is challenged. This includes, for example, glass formation and gelation of dense polymeric and colloidal systems (Guo *et al.*, 2012 ▸; Kwaśniewski *et al.*, 2014 ▸; Conrad *et al.*, 2015 ▸; Zhang *et al.*, 2017 ▸) or soft matter under shear (Leheny *et al.*, 2015 ▸).

The exceptional high degree of coherence in the hard X-ray regime (Grübel *et al.*, 2007 ▸; Gutt *et al.*, 2012 ▸; Lehmkühler *et al.*, 2014 ▸) makes FEL facilities promising instruments for XPCS studies. Here, the timescales accessible in sequential-mode XPCS are typically limited by the repetition rate of the FEL, *i.e.* at minimum 8.3 ms (LCLS; 120 Hz) at currently operating FEL sources (Carnis *et al.*, 2014 ▸; Lehmkühler *et al.*, 2015 ▸). This will be extended to the (sub)µs regime by the European XFEL (Tschentscher *et al.*, 2017 ▸). On the other hand, XFEL sources enable, for the first time, studies of dynamics in the range of fs to ns using split-pulse techniques or machine-based double shots (Roseker *et al.*, 2009 ▸; Sun *et al.*, 2018 ▸). Split-and-delay devices are currently under design or commissioning at most FEL facilities (Roseker *et al.*, 2012 ▸; Lu *et al.*, 2016 ▸; Zhu *et al.*, 2017 ▸; Hirano *et al.*, 2018 ▸). Recently, the first split-and-delay experiment has been demonstrated for studying the diffusion of nanoparticles in the nanosecond regime (Roseker *et al.*, 2018 ▸). Dynamics in the femtoseconds regime are uniquely studied by X-ray speckle visibility spectroscopy (XSVS) as recently performed on liquid water (Perakis *et al.*, 2018 ▸). Therein, the pulse length is modified and the dynamics are accessed *via* the change of speckle contrast as a function of exposure time. In addition, XSVS has been demonstrated as a promising technique for low-dose experiments at storage-ring sources (Verwohlt *et al.*, 2018 ▸).

For XPCS experiments at FEL facilities at least two pulses with a delay τ hitting the same sample spot are necessary to determine the dynamics. Therefore, special attention has to be paid to the influence of radiation damage. This, in particular, is essential for soft-matter systems. In our recent study at SACLA we demonstrated that XPCS can indeed be performed on a prototypical soft-matter sample, *i.e.* the diffusion dynamics of silica nanoparticles in suspension (Lehmkühler *et al.*, 2015 ▸). Apart from primary beam damage, for example ionization or destruction of bonds, exposure of X-rays may lead to a heterogeneous heating of the sample that may influence its dynamics dramatically (Grübel *et al.*, 2007 ▸; Carnis *et al.*, 2014 ▸; Lehmkühler *et al.*, 2015 ▸).

Nevertheless, pulsed sources can be an advantage for studying radiation-sensitive samples. They enable the comparison of data taken from fresh sample spots that are unaffected by radiation-induced damage due to the ultrafast exposure, with data taken from spots that have experienced X-ray exposure before, which is an unavoidable consequence of sequential-mode XPCS. In this article, we study the dynamics of radiation-sensitive colloidal systems at the SACLA FEL. As samples we chose two different dense colloidal PNIPAM [poly(*N*-isopropylacrylamide)] systems. Since these materials scatter X-rays weakly, experiments below the radiation-damage threshold at storage-ring sources are challenging. After determination of the threshold before radiation damage sets in, the XPCS analysis was performed on ensembles of speckle patterns taken from both samples. We observed sub-diffusive dynamics that show different length scale dependencies for core-shell systems and a PNIPAM nanogel.

## 2. Experimental   

### 2.1. XPCS analysis   

In XPCS experiments, the sample dynamics are studied by the time-correlation function 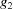 of the scattered intensity 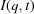 at the modulus of the wavevector transfer *q* and time *t*, *via*

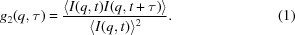
Here, 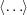 denotes an ensemble average over all equivalent times *t* and detector pixels contained in a chosen wavevector range 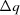. τ is the lag time that is defined by multiples of the repetition rate at FEL sources. The correlation function 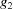 can be expressed by the modulus of the intermediate-scattering function 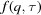 using the Siegert relation 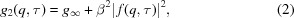
where β is the speckle contrast and 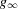 the baseline, which equals 1 for ergodic systems. The speckle contrast is usually setup-dependent and is constant during experimental runs at storage-ring sources by fixing beam size and coherence lengths. However, for SASE-FEL, shot-to-shot fluctuations of intensity, pointing and coherence result in an effective reduction of contrast in 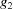 functions (Carnis *et al.*, 2014 ▸; Lehmkühler *et al.*, 2015 ▸). In order to estimate the effective contrast in such experiments, we measure correlation functions of static samples for which equation (2) becomes independent from τ and reads 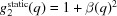.

For dynamics of soft-matter materials, the modulus of the intermediate-scattering function can be further expressed by a stretched or compressed exponential 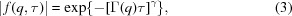
with the relaxation rate 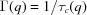 and relaxation time 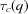. The *q* dependence of 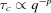 and the shape parameter γ provide information of the sample dynamics, such as diffusion (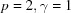), ballistic motion (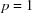) or cooperative dynamics (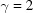) found in soft-matter glasses (Caronna *et al.*, 2008 ▸; Conrad *et al.*, 2015 ▸).

### 2.2. Samples   

We took a series of speckle patterns from three different sample systems. The first sample was a dried powder of poly(methyl-methacrylate) (PMMA) colloidal spheres (mean radius 125.5 nm). This was used as static sample to determine the speckle contrast as described in earlier publications (Lehmkühler *et al.*, 2014 ▸, 2015 ▸).

Samples 2 and 3 were radiation-sensitive suspensions based on poly(*N*-isopropylacrylamide), also known as PNIPAM. PNIPAM is frequently studied because of its volume phase transition from a swollen hydrated to a shrunken dehydrated state around 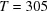 K (Das *et al.*, 2006 ▸). Like other polymers, PNIPAM is very sensitive to radiation damage and the scattering contrast difference between PNIPAM, in particular in hydrated states, and the solvent water is very weak. This challenges X-ray scattering and spectroscopy studies in general, and investigations on dynamics using XPCS in particular. In this article we want to address these problems with two different samples.

Sample 2 was an aqueous suspension of core-shell systems with silica particles as core (radius 41 nm with a size polydispersity of 0.08) and a PNIPAM shell of 40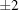 nm thickness at room temperatures. Details of the synthesis of such particles can be found in Nun *et al.* (2017 ▸). In the dehydrated state the PNIPAM shell shrank down to a thickness of about 5 nm, as probed by dynamic light scattering (DLS) on a dilute suspension. The particle concentration was about 11 wt%, which corresponds roughly to a volume fraction of 0.07 in the collapsed particle state above 313 K.

Sample 3 was a dispersion of PNIPAM nanogel particles. The particles were synthesized following the procedure described in Huang & Hu (2007 ▸). The sample was characterized by dynamic light scattering yielding a hydrodynamic radius of 121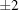 nm at 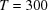 K. The concentration of PNIPAM was 12.7wt%, corresponding to an estimated volume fraction of 0.12 in the collapsed state.

### 2.3. Experimental setup at SACLA   

The experiment has been performed at beamline BL2 of the SACLA FEL (Japan) (Ishikawa *et al.*, 2012 ▸; Tono *et al.*, 2013 ▸; Yabashi *et al.*, 2015 ▸) in small-angle scattering geometry. X-ray pulses were generated with a repetition rate of 30 Hz. The photon energy was chosen to be 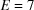 keV, with an energy resolution of 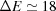 eV (pink beam). An average pulse energy of approximately 110 µJ was measured at the sample position, corresponding to about 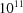 photons per pulse. The beam was focused by a Kirkpatrick–Baet mirror system reaching a spot size on the sample position of about 1.5 µm × 1.5 µm on average. Silicon and aluminium attenuators were used to limit the X-ray exposure resulting in a transmission value of 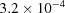. The samples had been filled into quartz capillaries of 0.7 mm diameter and 10 µm thick walls that were vacuum sealed afterwards and placed in the MAXIC chamber (Song *et al.*, 2014 ▸). Single-shot diffraction patterns were recorded by a dual MPCCD detector (Kameshima *et al.*, 2014 ▸) placed 3 m downstream of the sample. Diffraction patterns were taken in a series of 120 patterns (4 s total duration for each run) at 451 different spots on sample 2, 516 spots on sample 3, and a series of 1000 patterns at 5 spots for sample 1. The samples were studied at a temperature of 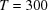 K where PNIPAM is in a swollen state, thus being a prototypical colloidal soft-sphere system.

## 3. Results and discussion   

**Figure 1:**
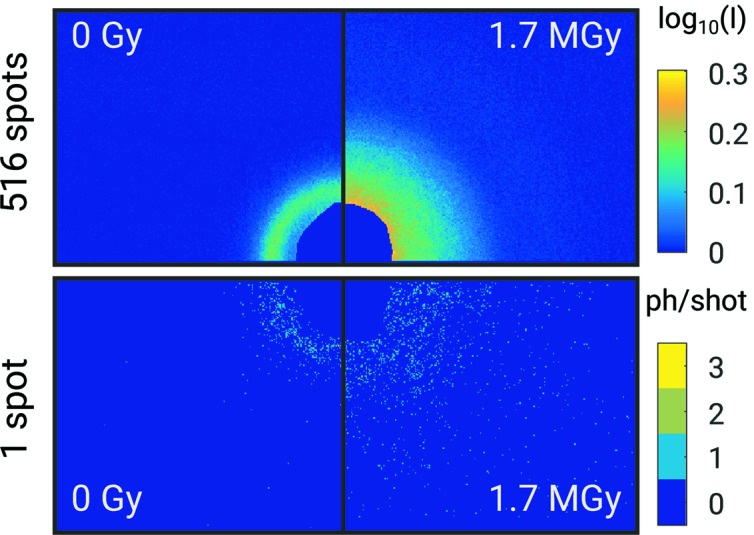
Diffraction patterns taken from PNIPAM. Top: averaged signals of 516 single shots on fresh sample spots (0 Gy) and after exposure to 119 shots corresponding to 1.7 MGy. Bottom: the same for single shots.

Firstly, the scattering signals from the PNIPAM samples were analyzed. Therefore, all patterns were summed up according to their position in the 120 shot series, *i.e.* sums of all first, second, third *etc.* patterns are calculated independently. Such averaged patterns can be associated to an average dose *D* that was exposed before the corresponding pattern had been taken. Thus, the first pattern was taken at 0 Gy, while those last in the series were recorded after a total dose of several MGy. This corresponds to 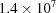 photons per µm^2^ for a single shot and about 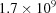 photons per µm^2^ for the total series of 120 single shots. Example diffraction patterns taken from the PNIPAM nanogel sample (sample 3) are shown in Fig. 1 ▸. Here, quadrants of diffraction patterns averaged over all spots on the sample are shown in the top part together with single-shot patterns in the bottom part. For single-shot patterns, a maximum intensity of 3 photons per pixel has been observed. Comparison of the averaged patterns reveals the effect of radiation damage. While the fresh sample shows a clear structure-factor ring, this signal is smeared out for 1.7 MGy and forward scattering increases.

**Figure 2:**
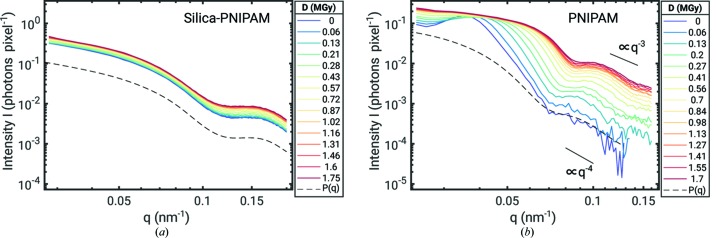
Azimuthally integrated intensity 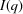 per shot from both samples for various doses: (*a*) silica-PNIPAM core-shell system. The dashed line (offset for clarity) corresponds to a form factor of a core-shell system consisting of 41 nm core radius of silica particles and 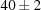 nm thick PNIPAM shell. (*b*) PNIPAM nanogel. The dashed line corresponds to a form factor of spheres with 60.5 nm radius.

The effect of the X-ray dose on the sample structure is highlighted in Fig. 2 ▸, where azimuthally integrated intensities 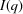 are shown. For silica-PNIPAM the scattering signal is dominated by the silica core. The main difference is an overall increase in scattering intensity with *D*, with a small change in shape of 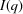. For the fresh sample, 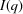 can be well described by a core-shell model shown as a dashed line in Fig. 2 ▸(*a*). For the PNIPAM nanogel particles, three characteristics of radiation damage can be observed with increasing *D*: (i) the first minimum originated from the particle’s form factor shifts to larger *q*, assuming shrinking of the particles, (ii) the *q* dependence in the Porod regime changes within the accessible *q* range from 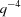 towards 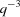 and (iii) the structure-factor peak visible at low *D* first broadens and shifts to larger *q* before it finally disappears. In addition, a form factor of spherical particles of radius 60.5 nm is shown in Fig. 2 ▸ by a dashed line. The particles appear to be about a factor of 2 smaller at such high concentrations than in the dilute limit measured by DLS. We connect this to a conformational change at high concentrations studied here compared with dilute particles. In addition, the hydrodynamic radius measured in DLS experiments is typically larger than the radius obtained in SAXS, especially for brush-like structures as in the case of the PNIPAM nanogel. It is important to note, that we do not see indications of particle agglomeration as often reported in radiation-damage studies on biological macromolecules (Jeffries *et al.*, 2015 ▸; Hopkins & Thorne, 2016 ▸; Schroer *et al.*, 2018 ▸).

**Figure 3:**
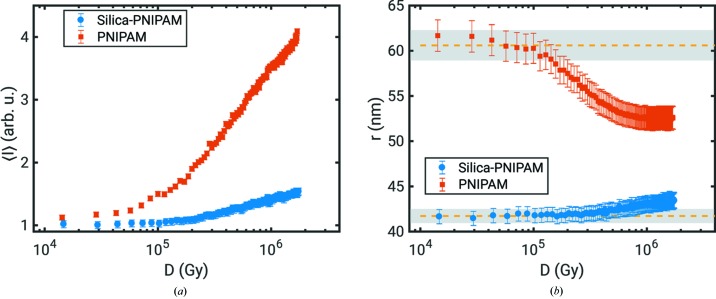
Effect of radiation damage on (*a*) the averaged intensity 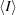 normalized to the measurement at 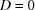 Gy and (*b*) the particle radius *r* for both samples. The dashed lines in the bottom part mark the radius obtained from the first patterns of the series, *i.e.* for 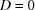 Gy, the grey areas mark the error bar of those values.

More details of the radiation damage for sample 2 and 3 are shown in Fig. 3 ▸. Therein, the intensity averaged over the *q* range between 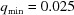 nm^−1^ and 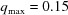 nm^−1^ is shown in Fig. 3 ▸(*a*) as a typical parameter qualifying radiation damage (Hopkins & Thorne, 2016 ▸). The values are normalized to the first patterns measured at 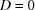 Gy. A clear increase of scattering intensity with *D* becomes visible which is less dominant for the silica-PNIPAM sample. Fig. 3 ▸(*b*) shows the particle radius as function of *D*. Since a reliable form-factor fit to the data is challenging because of the limited *q* range and high volume fraction, in particular at low *D*, the radius *r* was estimated from the position of the first minimum of the form-factor oscillations. Therefore, the curves were averaged, smoothed and corrected for the 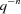 dependence before obtaining the minimum 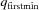. The radius is given by 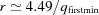 for spherical particles (Jeu, 2016 ▸). Here, both samples show a different behaviour. While the size of the PNIPAM spheres (sample 3) gets smaller with *D*, the radius of the core-shell systems (sample 2) effectively increases. We attribute this to the different nature of both samples. For sample 3, these radiation-induced effects may originate from the radiolysis of the solvent water, which is commonly observed in radiation-damage studies (Jeffries *et al.*, 2015 ▸). As the conformational state of PNIPAM is very sensitive to changing solvent properties (Schroer *et al.*, 2016 ▸) the particles seem to collapse as reflected by the reduced radius. Furthermore, their concentration increases locally as expressed by the increase in scattering intensity. This effect can also be driven by a change of scattering contrast between the particles and water. Finally, the potential change in particle size, shape and concentration leads to a different structure that modulates the structure factor. Furthermore, heating above 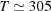
 K caused by X-ray exposure may result in a collapse of the particles which promotes formation of a particle network, thus increasing scattering intensity at small *q*. In contrast, the silica core of sample 2 is rather insensitive to radiation (Lehmkühler *et al.*, 2015 ▸). At low *D*, scattering is dominated by the core (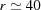 nm) whose density contrast to water is more than a factor of ten larger than for the PNIPAM shell. The increase in the particle size is thus interpreted to arise from a densification of the damaged PNIPAM shell at high *D*. Furthermore, following the discussions in previous work (Hruszkewycz *et al.*, 2012 ▸; Carnis *et al.*, 2014 ▸; Lehmkühler *et al.*, 2015 ▸), we estimated the effect of beam-induced heating of the samples. Considering the average photon density per shot of 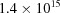
 photons cm^−2^ and tabulated values for silica and PNIPAM for mass attention, density and heat capacity, about 48 photons are absorbed by the silica core and 1.5 photons by the PNIPAM shell of sample 2. Likewise, about 3.5 photons are absorbed by the PNIPAM particles of sample 3. Assuming that all energy is converted to heat, we obtain a maximum temperature increase of 122 K for the silica core and 8.5 K for the PNIPAM shell as well as 9 K for the PNIPAM particles. These values are below those reached in previous studies of soft-matter materials (Carnis *et al.*, 2014 ▸; Lehmkühler *et al.*, 2015 ▸), and, most importantly, this heat increase relaxes within a few µs, well before the next shot impinges on the sample.

Both parameters 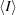 and *r* were used to identify a maximum dose that is tolerable for the XPCS analysis. Especially taking the variation of *r* into account, we found 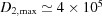 Gy for the silica-PNIPAM core-shell sample and 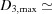 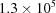 Gy for PNIPAM, corresponding to a series of 27 and 9 patterns, respectively. These results are in line with studies on biological macromolecules and ionic liquids, where thresholds between a few to several 100 kGy are observed (Jeffries *et al.*, 2015 ▸; Verwohlt *et al.*, 2018 ▸). Similar to those studies, we used structural parameters to define the threshold for studying dynamics. While this procedure has been successfully used in soft-matter systems over decades, recent studies on network glasses showed reversible beam-induced effects on sample dynamics at atomic length scale without any structural change (Ruta *et al.*, 2017 ▸). However, because of the reversibility of these effects they cannot be assigned as radiation damage as commonly understood. The XPCS analysis has been performed for all spots for the corresponding samples independently below the radiation-damage threshold. The results were averaged afterwards over all studied spots.

**Figure 4:**
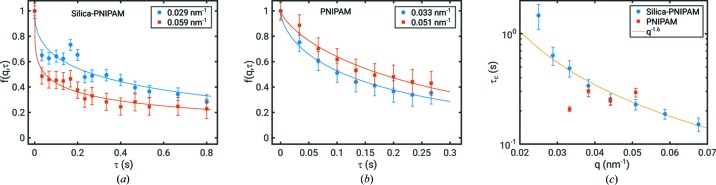
Dynamics results. (*a*) and (*b*): intermediate scattering functions for both samples at two *q* values. The solid lines are fits of equation (3). (*c*) Relaxation time 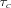 as function of *q*. The solid line shows a fit of 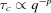, yielding 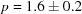 for silica-PNIPAM.

Results of the XPCS analysis are shown in Fig. 4 ▸. Here, moduli of the intermediate scattering function 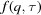 are shown at two *q* values for both samples. Because of the shot-to-shot fluctuations, we additionally add a point at 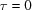 s that is obtained from the analysis of the static sample (Lehmkühler *et al.*, 2015 ▸). Here, we found a contrast of the correlation function of 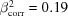. This is below the contrast observed in single shots (Lehmkühler *et al.*, 2014 ▸) but slightly larger compared with our previous experiment (Lehmkühler *et al.*, 2015 ▸). This can be explained by the different setup and operation conditions. The intermediate-scattering functions were obtained by equation (2) using 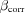 for normalization. Afterwards, the obtained 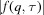 were modelled following equation (3). The best fits to the data are given by solid lines in Fig. 4 ▸. The relaxation times 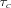 are shown in Fig. 4 ▸ (*c*). The relaxation times due to Brownian motion in the dilute limit would be in the region of 0.01 to 0.1 ms at the given *q* range for the samples, demonstrating slowing down at the high volume fractions studied here. Because of the higher count-rate of the silica-PNIPAM sample, the *q* dependence of 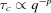 could be modelled with an exponent 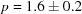 which corresponds to an intermediate between ballistic (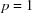) and diffusive (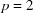) motion. In contrast, only four *q* values provided reliable fits for PNIPAM, showing almost no *q* dependence. This weak *q* dependence may indicate stress-dominated dynamics, however, the statistics are too low to draw a final conclusion at this point. In addition, since 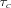 could only be accessed in the vicinity of the structure-factor peak, it may be dominated by de Gennes narrowing (de Gennes, 1959 ▸; Grübel *et al.*, 2008 ▸), *i.e.* slowing down of the dynamics at the next-neighbour distance. The shape exponent was found to be 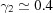 and 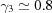 with small variations with *q* for samples 2 and 3, respectively. Exponents of 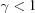 are connected to a distribution of relaxation times that is broader than a Gaussian which indicates sub-diffusive and heterogeneous dynamics. As the relaxation times are more than three orders of magnitude slower than expected for diffusive dynamics, we conclude that they are dominated by particle–particle interactions. Thus, an effect of size and shape polydispersity on the dynamics that leads dynamical heterogeneities with 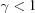 as well (Andrews *et al.*, 2018 ▸) can be neglected. The results are in qualitative agreement with various XPCS studies on polymeric systems where stretched exponentials together with 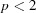 suggesting super-diffusive dynamics are reported frequently (Nogales & Fluerasu, 2016 ▸; Hoshino *et al.*, 2013 ▸; Ruta *et al.*, 2014 ▸; Conrad *et al.*, 2015 ▸; Senses *et al.*, 2017 ▸).

In conclusion, we present an XPCS study at a hard X-ray FEL on radiation-sensitive soft polymeric colloids. While most studies on polymer-dominated dynamics are performed using tracer particles, we were able to study nanoscopic polymer particles directly. We obtained dynamics information from concentrated sample systems, *i.e.* a silica-PNIPAM core-shell system as well as a PNIPAM nanogel, by carefully analysing the threshold for radiation damage on structural parameters. Because of the ultrashort pulse lengths, we were able to determine the effects of radiation damage by comparing the data with results that did not experience beam damage, *i.e.* the first patterns of the series. Using FEL shots with a repetition rate of 30 Hz, effects of beam-induced heating did not affect the data and thus are likely to only play a minor role, if any. SASE fluctuations were taken into account by measuring the contrast of speckle patterns from static samples. The extracted dynamics indicate non-diffusive dynamics for both systems, suggesting ballistic and stress-dominated particle motion. In addition, the stretched shape of the correlation functions may indicate dynamical heterogeneities. These results demonstrate the feasibility of performing scattering experiments on soft-matter materials at FEL sources. In particular, the next-generation FEL facilities such as the European XFEL will provide access to (sub-)µs structural dynamics on (sub-)nm length scales, unreached by other techniques so far. At these time scales many processes of soft matter can be observed such as protein folding (Freddolino *et al.*, 2010 ▸) or diffusion of nanometre systems in aqueous environments. XPCS at FEL facilities would be the technique of choice to answer these fundamental questions.
